# Points Worth Knowing

**Published:** 1895-04

**Authors:** 


### POINTS WORTH KNOWING.

---

The tongue of a giraffe is nearly eighteen inches long.

The average strength of a horse is seven and one-half times greater than a man.

Opossums are said to be the only animals that make a more elaborate toilet than cats.

The wild tamarind when eaten by horses is said to be followed by a loss of hair from the mane and tail.

An examination under the State Board of Veterinary Medical Examiners was held at Columbus, Ohio, on April 2d.

